# Engagement of Urology Faculty With the Social Media Accounts of Residency Applicants: Does #UroSoMe Influence the Match?

**DOI:** 10.7759/cureus.107907

**Published:** 2026-04-28

**Authors:** Rachel Vancavage, Seamus Barrett, Paul Feustel, Barry Kogan, Brian M Inouye

**Affiliations:** 1 Department of Urology, Albany Medical College, Albany, USA; 2 Department of Neuroscience and Experimental Therapeutics, Albany Medical College, Albany, USA

**Keywords:** interview, residency, social media, twitter, urology match, x

## Abstract

Introduction: Social media provides urologists a platform to network and share academic insights. Previous studies have suggested that Twitter (X; X Corp, Bastrop, TX, USA) specifically plays a role in gathering information about programs for applicants but does not influence rank list decisions. Our study examines the role of Twitter (X) among residency programs in candidate selection for interviews and rank list decisions. Materials and methods: We developed a survey and distributed it via email to faculty members at all 151 Accreditation Council for Graduate Medical Education-accredited urology residency programs, as well as at the Society of Academic Urologists meeting at a previous annual American Urological Association conference. Results: We obtained 70 anonymous responses regarding faculty usage and interactions with applicants on Twitter (X). Of those who responded, 70% have a Twitter (X) account, and of those, 73% use Twitter (X) at least weekly. While most faculty members on Twitter (X) do not use social media to gather information about applicants before interviewing them, some respondents do. Although Twitter (X) usage did not have a statistically significant effect on interview offers or rank lists, 10% of respondents reported changing their rank lists based on Twitter (X). Conclusions: Applicants, whether they directly interact with programs or not, should be advised to maintain a high degree of professionalism on social media and remain vigilant, knowing the potential impact.

## Introduction

Social media in urology plays an important role in expanding the body of knowledge by providing urologists with a platform for easy networking and the sharing of academic insights [1-3]. Twitter (now known as "X"; X Corp, Bastrop, TX, USA) specifically has gained popularity for these academic opportunities. More recently, the use of social media in the urology residency application process has been expanding. One recent study found that, from the applicant's perspective, Twitter (X) plays an important role in gathering program information but does not impact applicant rank list decisions or outcomes [4]. Previously, the use of social media as an adjunct to support one’s job application (e.g., for residency) was limited due to concerns about professionalism [5,6].

Twitter (X) is accessible by both applicants and programs, yet little is known about the program’s use of Twitter (X). Since Twitter (X) is personalized, theoretically, a program could use an individual's account to gain additional insight into an applicant, leading to an altered ranking, whether better or worse. For instance, in a since-retracted publication, a group of medical professionals stated that social media profiles that they deemed unprofessional would deter patients [7]. These beliefs could subconsciously affect rankings. Given the limited information about how programs use Twitter (X), we surveyed the Society of Academic Urologists (SAU) program directors and faculty on this topic. We hypothesized that faculty who have interacted on Twitter (X) with applicants will be influenced by an applicant’s social media to change rank list or interview invitation decisions.

This article was previously presented as a meeting poster at the 75th Annual Meeting of the Northeastern Section of the American Urological Association (AUA) on October 23, 2023.

## Materials and methods

We developed an anonymous 26-item multiple-choice survey and sent this to all residency program coordinators to distribute to the faculty of all 151 Accreditation Council for Graduate Medical Education-accredited urology programs. Residency coordinators were asked to distribute the survey to their faculty. The survey was not additionally publicized (e.g., on Twitter (X)) to protect from outside responses. The variables we investigated include demographics, frequency and quality of Twitter (X) use, types of interactions with candidates, and faculty perspectives on candidates' Twitter (X) use. Data are expressed as frequencies and percentages, and associations are tested using Fisher’s exact test. The Albany Medical College Institutional Review Board issued approval 6629.

## Results

A total of 70 SAU members completed our survey. Seventy percent of respondents reported having a Twitter (X) account. Most respondents with Twitter (X) accounts (73%) use the application between daily and weekly. Of those who endorsed having a Twitter (X) account, 34 respondents (70%) reported identifying a urology applicant on the app. Of these 34 respondents who identified urology applicants, 11 respondents (33%) stated that they had further investigated the tweets of that individual. Twitter (X) user or not, 29% of respondents reported seeing something they deemed unprofessional on an applicant's social media account, 68% of which involved an unprofessional picture.

**Figure 1:**
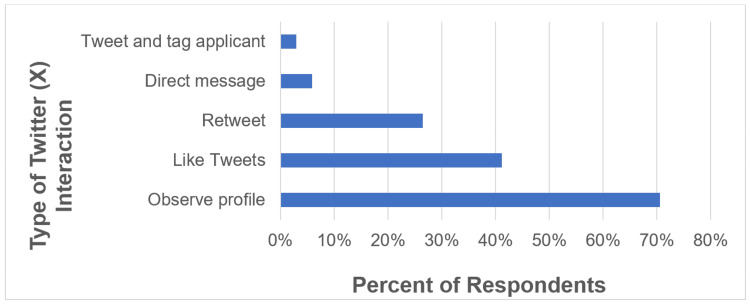
Types of Twitter (X) interactions

Among the 49 surveyed Twitter (X) users, 33% reported interacting with a urology applicant on Twitter (X). The majority of those who interact do so by “clicking on” an applicant’s profile to observe rather than by direct communication (Figure 1). There was no association between interacting with an applicant on Twitter (X) and changing an interview invite offer (p = 0.67) or changing the rank list based on social media (p = 0.35).

When considered in the context of our primary hypothesis, six (12%) of these faculty respondents with Twitter (X) accounts reported changing interview-invite and/or rank-list decisions based on social media findings. Five of six (83%) of these respondents answered “yes” to both questions; however, one (17%) reported that they had changed the interview invite but not the rank list decision. Heard et al. found that urology program directors believe applicants should avoid discussing politics, drugs, alcohol, and other polarizing topics on social media. Yet, a very low percentage reported that applicants' social media activity had harmed their chances of matching [8]. We find it interesting that six respondents changed their decision based on an applicant’s Twitter (X), suggesting that social media content may be of varying importance to different faculty members. Our survey did not ask whether the respondent’s change in rank list or interview invite was beneficial or detrimental to the applicant, so we can only conclude that social media had some influence. Overall, our data suggest that some urology applicants are posting content deemed unprofessional, though it is not so offensive as to negatively influence their chances of matching. Still, urology candidates should be cautious with what content they post, as a third of the faculty respondents endorse engaging in some form of interaction with applicants.

## Discussion

Our findings are similar to a survey from the 2019-2020 AUA match cycle that examined applicants' and programs' perspectives on important application attributes, in which most applicants thought their social media profiles were weighted more heavily. In contrast, 6% of faculty reported reviewing an applicant’s social media presence before extending an interview invitation [9]. That survey was conducted before the COVID-19 pandemic and the implementation of virtual interviews. Our data suggests that Twitter (X) presence for applicants is unlikely to change their likelihood of matching in the setting of virtual interviews post-pandemic, though this finding is based on a small sample and represents a minority of respondents. It seems that faculty have not changed their attitude regarding the use of social media, even though there have been multiple post-COVID-19 virtual match cycles. Now that the SAU has allowed for in-person, hybrid, and virtual interviews, it is unclear if the utility of social media in the match process will change. In the future, we hope to expand our survey to examine this concept.

In our present study, a response rate cannot be calculated because the SAU required that survey invitations be sent to each program coordinator, who, in turn, was asked to distribute them to their faculty. We therefore do not know how many programs distributed the survey or whether it reached as many participants as we intended. As a result of our distribution plan, we may have ended up with fewer total respondents. This, therefore, limits the study's statistical power and increases the likelihood of type II error. Due to the survey's anonymity, we cannot determine whether multiple responses originated from the same institution, which could have skewed the data. It is also possible that bias may be introduced, as those who chose to respond may value social media more than those who did not. Further, some faculty may not wish others to know the extent of their social media usage, even if the survey is anonymous. We think this is likely to be limited, but we have no way of assessing that at this point. The anonymity and nature of our survey questions also prevent the analysis of posts that influenced faculty rank list and interview offer decisions. Further iterations of this study could include questions that specify post category, such as whether they are personal, religious, or political in nature, and their effects on decisions. Lastly, the study design does not permit inference about the direction of social media influence on the conclusions. Future directions of our research hope to examine this by possibly using a validated questionnaire and gathering a response rate.

## Conclusions

An applicant’s presence on Twitter (X) did not have a statistically significant impact on their likelihood of interviewing or on their rank list in our study. Although not statistically significant, 10% of the faculty reported that Twitter (X) findings affected their applicant rankings. This suggests that applicants should be aware that any controversial or potentially unprofessional posts during the application process may be under scrutiny, and they should therefore maintain a high degree of professionalism on social media.

## Appendices

Survery

1. What is your AUA geographic section?

a. Mid-Atlantic Section

b. New England Section

c. New York Section

d. North Central Section

e. Northeastern Section

f. South Central Section

g. Southeastern Section

h. Western Section

2. I am a:

a. Academic Faculty Member

b. Associate Program Director

c. Associate/Assistant Program Director

d. Department Chair, Division Chief

e. Member of SAU, neither chair nor program director

f. Residency Program Director

3. Age

a. <35 years old

b. 36-50 years old

c. 51-65 years old

d. >65 years old

4. Gender

a. Female

b. Male

5. Do you have a Twitter account?

a. Yes

b. No

6. How frequently do you access Twitter

a. Less than weekly

b. Between daily and weekly

c. At least once a day

7. Through posts or examining a profile, have you identified a Twitter user as a urology applicant over the last year?

a. No

b. Yes

8. If you have identified a Twitter user as a potential urology applicant, have you ever looked into previous Tweets of that profile?

a. No

b. Yes

9. How do you interact with urology applicants on Twitter? (Select all that apply)

a. I do not use Twitter

b. I do not interact with urology applicants

c. Like their tweets

d. Retweet their post

e. Tweet and tag applicant

f. Direct message

g. Observe profile but do not like, retweet, or direct message

10. Have you interacted (liked a tweet, retweeted a post, direct messaged, tagged) with a urology applicant on Twitter in the last year?

a. No

b. Yes

11. If you interacted with an applicant within the last year, did you interact with urology applicants on Twitter before COVID-19?

a. Yes

b. No

c. N/A, I did not have Twitter prior to the COVID pandemic (March 2020)

d. N/A, I have not interacted with an applicant over the last year

e. N/A, I do not currently have Twitter

12. When do you interact with urology applicants on Twitter? (Select all that apply)

a. Anytime

b. During academic conferences

c. Between urology application cycle (February-September)

d. During application cycle (October-January)

13. Do you use social media to intentionally gain information about which urology applicants you may want to interview?

a. No

b. Yes

14. If you have identified a Twitter user as a potential urology applicant, have you ever looked into other social media (Facebook, Instagram, TikTok, YouTube) of that applicant if they DO have linkable accounts?

a. No

b. Yes

15. If you have identified a Twitter user as a potential urology applicant, have you looked into other social media (Facebook, Instagram, TikTok, YouTube) of that applicant if they DO NOT have linkable accounts?

a. No

b. Yes

16. Have you ever seen anything on a potential applicant's social media presence that you have deemed as unprofessional?

a. No

b. Yes

17. If you have found an applicant's post to be unprofessional, which aspect made you think so? (Select all that apply)

a. Picture of an applicant

b. Endorsement of a belief

c. Linguistic (e.g., misspelling, poor grammar)

d. Use of profanity

e. Other

18. After observing an applicant on Twitter, did that change whether or not you would offer them an interview?

a. No

b. Yes

19. After observing an applicant on Twitter, has that changed how you ranked them?

a. No

b. Yes

20. Has a urology applicant reached out to you on Twitter since March 2020?

a. No

b. Yes

21. If a urology applicant did reach out to you, how did they do so? (Select all that apply)

a. Direct messaged you

b. Liked your Tweet

c. Commented on your Tweet

d. Retweeted your post

22. If a urology applicant did reach out to you, how did they do so? (Select all that apply)

a. Interview advice

b. Institution query

c. Personal topic

d. Discussion of posted research topic

e. Other

23. Has an applicant ever communicated with you about a non-professional topic?

a. No

b. Yes

24. Do you think having a Twitter presence is beneficial for a urology applicant?

a. No

b. Yes

25. What type of urology applicant do you think having a Twitter presence is important for? (Select all that apply)

a. Any applicant

b. Applicant without home program

c. Underrepresented minority applicant

d. Applicant who wishes to promote research

e. Applicant who wishes to promote themselves personally

f. Twitter is not helpful for applicants

26. From the perspective of faculty, what is the overall benefit of Twitter for urology applicants? (Select all that apply)

a. Networking

b. Promoting research

c. Promoting themselves for programs

d. Learning about Urology programs

e. Learning about surgical techniques

f. Discussing interest in Urology programs with that program's leadership

g. Learning about Urology in general

h. Other
